# Clinical efficacy of efgartigimod combined with intravenous methylprednisolone in the acute phase of neuromyelitis optica spectrum disorders

**DOI:** 10.1186/s13023-024-03501-6

**Published:** 2024-12-21

**Authors:** Wenjing Yang, Pei Chen, Jiaxuan Guo, Huiyu Feng, Xin Huang

**Affiliations:** 1 Department of Neurology, The First Affiliated Hospital, Guangdong Provincial Key Laboratory of Diagnosis and Treatment of Major Neurological Diseases, National Key Clinical Department and Key Discipline of Neurology, Sun Yat-Sen University, No. 58 Zhongshan Road 2, Guangzhou, 510080 China; 2 Guangxi Hospital Division of The First Affiliated Hospital, Sun Yat-Sen University, Nanning, China

**Keywords:** Neuromyelitis optica spectrum disorders, Efgartigimod, Intravenous methylprednisolone, Treatment of acute phase

## Abstract

**Background:**

Neuromyelitis Optica Spectrum Disorders (NMOSD) comprise a group of autoimmune-mediated, inflammatory, demyelinating central nervous system diseases caused by aquaporin-4 (AQP4) IgG autoantibodies. Efgartigimod is a human IgG Fc fragment that reduces antibody titers by targeting the neonatal Fc receptor (FcRn). This study documents the efficacy of efgartigimod combined with intravenous methylprednisolone (IVMP) in the acute phase of NMOSD.

**Methods:**

In this retrospective study, the medical records of NMOSD patients with acute attack who received efgartigimod plus IVMP or IVMP were reviewed. Treatment efficacy was assessed by the Expanded Disability Scale Score (EDSS) before and one month after treatment. Any side effects that occurred during the treatment period were recorded.

**Results:**

This study was performed on 11 patients (efgartigimod plus IVMP group [n = 4] and IVMP group [n = 7]). Efgartigimod plus IVMP was effective and had a satisfactory safety profile. EDSS was reduced by 0.5 ± 0.32 compared with the IVMP group (0.27 ± 0.02). Immunoglobulin was decreased in three patients, and the immunoglobulin G (IgG) levels gradually increased approximately 8 weeks after the last administration. Hyperlipidemia and elevated white blood cell count were common side effects. No infections or deaths occurred.

**Conclusions:**

Efgartigimod plus IVMP treatment is safe and well-tolerated in patients with acute-phase NMOSD.

## Background

Neuromyelitis Optica Spectrum Disease (NMOSD) is an autoimmune-mediated demyelinating disease of the central nervous system (CNS) with core clinical features of acute optic neuritis, acute myelitis, area postrema syndrome, acute mesencephalic syndrome, and cerebral syndrome [1]. Pathogenesis of NMOSD is associated with aquaporin-4 (AQP4) immunoglobulin G (IgG) [2], which targets AQP4 on the surface of CNS astrocytes and triggers the onset of inflammatory response resulting in CNS myelin loss [3, 4]. The prognosis for NMOSD is poor with irreversible disabilities if left untreated or when treatment is delayed at the acute phase. Early initiation of effective treatment is crucial to minimize CNS damage and the extent of disabilities. High-dose intravenous methylprednisolone (IVMP) is typically recommended as the primary treatment for acute NMOSD [5]. However, IVMP was ineffective in 16.2% patients, and only 17% showed complete remission [6]. Add-on therapies such as plasma exchange (PLEX), immunoadsorption (IA), and intravenous immunoglobulin (IVIG) are other strategies to eliminate the AQP4 IgG in the acute phase. However, side effects such as hemodynamic instability, allergic reactions, and shortage of blood products pose challenges in clinical practice [7]. Conventional treatments have suboptimal results and drawbacks. Newer drugs are focused on antigen–antibody binding, AQP4 IgG antibodies, and lymphocyte activation with the aim to contribute to new therapeutic strategies in acute NMOSD.

The neonatal Fc receptor (FcRn) of IgG protects the immunoglobulin molecule from lysosomal catabolism by interacting with the Fc segment of IgG, resulting in the half-life of IgG being prolonged and a high concentration in the circulation [8, 9]. Therefore, blocking IgG and FcRn interactions holds considerable promise for rapid IgG clearance and degradation. This concept has substantial therapeutic applicability in IgG-mediated autoimmune diseases such as myasthenia gravis, chronic inflammatory demyelinating polyneuropathy, and NMOSD [10]. In an open-label, dose-escalation phase 1b study, the Expanded Disability Scale Score (EDSS) was reduced by 1.3 ± 0.4 at week 4 (baseline: 4.0 ± 1.0; week 4: 2.7 ± 1.3) in the 680-mg batoclimab (FcRn antagonist) group combined with IVMP [11]. FcRn antagonist with IVMP is safe and tolerated by NMOSD patients.

Efgartigimod is a human IgG1 Fc fragment modified using the Abdeg technology at five residues to increase its affinity for FcRn; it also has a higher affinity at acidic pH than near-neutral pH, resulting in the lysosomal degradation of unbound IgG [9, 12, 13]. Several clinical studies have shown substantial efficacy and safety of efgartigimod in IgG-mediated autoimmune diseases [14–16].

However, the efficacy of efgartigimod therapy for acute NMOSD remains poorly understood. Specifically, efgartigimod in combination with IVMP may be a conceivable strategy, but its clinical effectiveness in NMOSD has rarely been investigated. This study evaluates the efficacy of efgartigimod with concurrent IVMP in acute NMOSD attacks.

## Methods

The medical records of all NMOSD patients with acute attacks who were treated with efgartigimod and IVMP or IVMP at The First Affiliated Hospital, Sun Yat-sen University between September 2023 and October 2024 were reviewed. The inclusion criteria were as follows: (i) patients who met the 2015 International Consensus diagnostic criteria for NMOSD[1]; (ii) those who were seropositive for AQP4-IgG; (iii) those who experienced a relapse (relapse refers to the occurrence of new symptoms or exacerbation of original symptoms that last for ≥ 24 h and often occurs more than 30 days after the last attack without other recognizable causes such as fever or infection) [7]; and (iv) those who received efgartigimod concurrent with IVMP or IVMP at acute attacks.

This study was approved by the local institutional review board, and identifiable information was removed from the database records according to the data protection required by the ethics committee of the First Affiliated Hospital of Sun Yat-Sen University.

We analyzed the medical records including demographic (age and sex) and clinical characteristics, IVMP and efgartigimod use (dosage, treatment duration), AQP4 IgG titers, EDSS, and adverse events. The therapeutic effect was evaluated by the EDSS score before treatment and one month after treatment. Study data were summarized using descriptive statistics. Descriptive statistics were presented as a number, mean and standard deviation, and median (minimum and maximum) for quantitative data and number and percentage for qualitative data.

## Results

### Demographic and clinical characteristics

Between September 2023 and September 2024, four patients were treated with efgartigimod plus IVMP, and seven patients were treated with IVMP, during the acute period. All patients were female, aged 46–59 years (group 1) and 30–70 years (group 2). All subjects had acute attacks including area postrema syndrome (n = 1), myelitis (n = 1), and optic neuritis (n = 2) in group 1 and acute brainstem syndrome (n = 1), myelitis (n = 2), optic neuritis (n = 3), and acute brainstem syndrome (n = 1) in group 2. One patient had an EDSS of 7.0 at the time of the attack, and the remaining had EDSS of 2–4 (group 1) and 2–4.5 (group 2). Table 1 presents the clinical and demographic features and treatment details of all 11 patients. Before treatment, the average EDSS was 3.88 (group 1) and 3.71 (group 2). Thus, neurological dysfunction and disease severity were comparable between the two groups.

**Table 1:** Demographic and baseline characteristics

Groups	Patients	Age at onset (years)	Sex	Clinical characteristics	Time to treatment from attack (days)	IVMP	Efgartigimod dose (mg)	Efgartigimod administration times	EDSS (attack)	AQP4 IgG titers in serum (Baseline)	Times of attack
1	1	49	F	Area postrema syndrome	42	1000 (5d)	800	2	4.5	1:10	1
	2	46	F	Myelitis	10	1000 (3d), 500 (3d), 250 (3d), 125 (3d)	800	2	3	1:100	1
	3	53	F	Optic neuritis (bilateral)	30	500 (3d),	1200	2	4	1:100	3
	4	59	F	Optic neuritis (bilateral)	10	500 (5d)	800	2	4	1:32	2
2	5	56	F	Optic neuritis (right)	25	1000 (5d)	NA	NA	3	1:32	1
	6	52	F	Acute brainstem syndrome	9	1000 (3d)	NA	NA	1.5	1:10	1
	7	50	F	Myelitis, area postrema syndrome	60	1000 (3d), 500 (3d), 250 (3d), 125 (3d)	NA	NA	2	1:32	1
	8	70	F	Optic neuritis (bilateral)	16	500 (3d), 250 (3d), 125 (3d)	NA	NA	4	1:32	3
	9	53	F	Myelitis	8	1000 (3d), 500 (3d), 250 (1d)	NA	NA	7	1:100	4
	10	39	F	Optic neuritis (right)	19	1000 (3d)	NA	NA	4	1:320	1
	11	30	F	Myelitis	25	500 (5d)	NA	NA	4.5	NA	4

Group 1: Patients treated with efgartigimod and IVMP; Group 2: Patients treated with IVMP. F: Female; IVMP: Intravenous methylprednisolone; NA: Not applicable

### Therapeutic efficacy

**Fig. 1:**
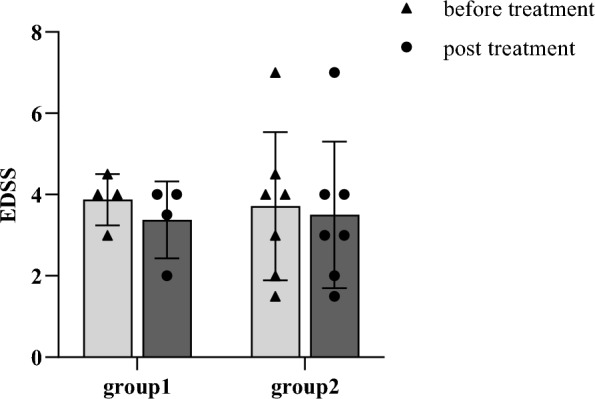
Changes in EDSS of the two groups

The changes in EDSS for each subject in the two groups before and after treatment are shown in Fig. 1 and supplementary materials_EDSS [17]. After one month, 2 (50%) patients in group 1 who received combination therapy experienced a decrease in EDSS scores. In contrast, only 1 (14.3%) patient in group 2 experienced a decrease in EDSS score. Post-treatment (1 month later), the average EDSS decreased by 0.5 ± 0.32 in group 1 and 0.27 ± 0.02 in group 2.

Within 1 month, two patients in group 1 experienced a decrease in their EDSS scores. One patient’s score decreased from 4.5 to 3.5, while the other’s score decreased from 3.0 to 2.5. Although there was no significant decrease in the EDSS for patient #3, her left-eye vision improved from blindness to hand movement at 50 cm.

In group 2, one patient (#6) had optic neuritis in the right eye. Before treatment, the visual acuity of counting fingers was at 30 cm; after treatment, it had essentially returned to normal. The EDSS decreased from 4.0 to 3.0. In addition, the EDSS of the six patients in group 2 remained unchanged.

### AQP4 – IgG titers and immunoglobulin levels monitoring

Before treatment for the acute attack, two patients (#2 and #3) of group 1 had an AQP4–IgG titer of 1:100, while the other two patients (#1 and #4) had titers of 1:10 and 1:32, respectively. After treatment, serum AQP4–IgG titers decreased in two patients (#1 and #3). On day 12, AQP4–IgG was undetectable in patient #1. On day 36, AQP4–IgG titers of patient #3 decreased from 1:100 to 1:32. On day 72, AQP4–IgG of patient #1 remained undetectable. The change of AQP4 – IgG titers in group 1 is summarized in Table S1 [17].

**Fig. 2:**
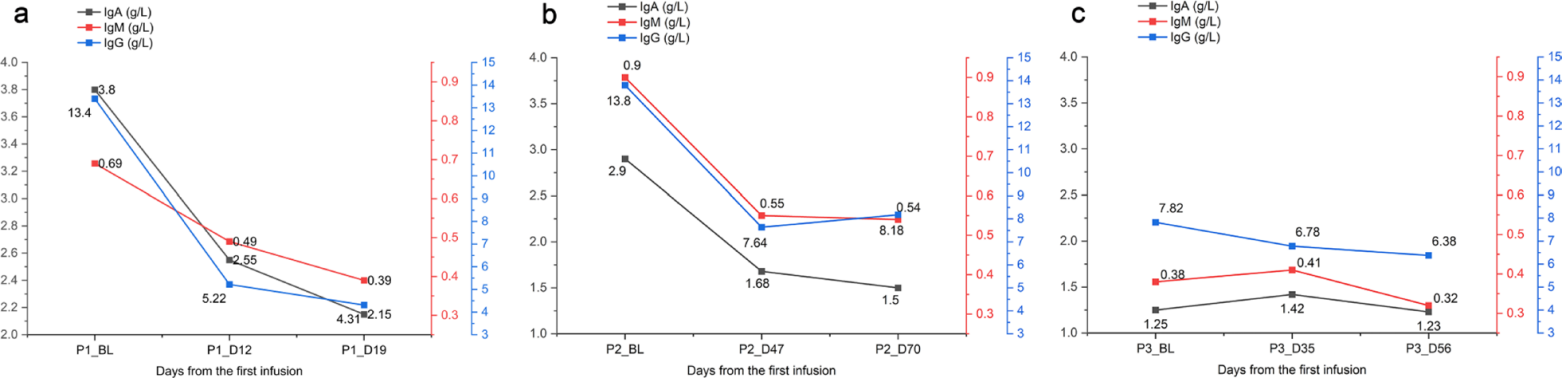
Changes of IgA, IgM, and IgG. **a** Patient’s (#1) serum IgG level decreased by 58.56% at 19 days. **b** Patient’s (#2) serum IgG level decreased by 44.64% 6 weeks later. **c** Serum IgG change of patient #3. Reference range: IgA 1.45–3.45 g/L; IgM 0.92–2.04 g/L; IgG 10.13–15.13 g/L. BL: baseline; Ig: immunoglobulin

Three patients’ (#1–3) immunoglobulin data were analyzed (Fig. 2). IgG in one patient decreased by 58.56% in 19 days, similar to the report by da Silva et al., where the median time to maximum response was 14–27 days following the first administration of efgartigimod [9]. IgG changes in two patients (#2 and #3) were consistent with the findings that the levels returned to near baseline approximately 8 weeks after the last dose of efgartigimod [18, 19].

### Adverse events

Adverse events are detailed in Table 2. No deaths or serious adverse events occurred in either group. Three patients (3/4) in group 1 and three patients (3/7) in group 2 experienced adverse events. The four patients tolerated the combination of efgartigimod plus IVMP well, and none of the patients discontinued efgartigimod or IVMP. Common side effects included hyperlipidemia and elevated white blood cell counts in both groups. No infections or serious infections were detected. Some patients exhibited elevated white blood cell counts without clinical symptoms, possibly linked to hematopoiesis induced by high-dose IVMP [11].

**Table 2:** Adverse events

Adverse events	Group1, n	Group2, n
Fatigue	0	0
Migraine	0	0
Nausea and vomiting	0	0
Elevated blood pressure	2	0
Hyperuricemia	0	0
Hyperlipidemia	2	2
Elevated intraocular pressure	1	0
Infection	0	0
Respiratory tract infection	0	0
Urinary tract infection	0	0
Tinea unguium	0	0
Other infections	0	0
Severe infection	0	0
Rash	0	0
Increased white blood cell count	2	3
Increased neutrophils count	2	2
Elevated C-reactive protein	0	0
Elevated transaminase levels	1	1

Group 1: Patients treated with efgartigimod and IVMP; Group 2: Patients treated with IVMP

## Discussion

The EDSS was improved in two patients (#1 and #2). The rapid improvement in acute symptoms was possibly related to efgartigimod combined with high-dose IVMP. Although the effect of a single efgartigimod treatment was not detectable, efgartigimod can act as an adjuvant therapy to IVMP with the ability to rapidly clear antibodies. Previous research showed a positive neurological effect of FcRn antagonist. Batoclimab, an FcRn antagonist, also showed an improvement in visual acuity and walking time in NMOSD patients with acute myelitis and/or optic neuritis in an open-label, dose-escalation phase 1b study [11]. Furthermore, a case report found that efgartigimod as a rescue following IVMP relieved serious vision loss with a satisfactory safety profile in a female patient with an acute NMOSD attack [20] In our study, patient #2—with AQP4 antibody titer 1:100—achieved a 1-point decrease in EDSS 1 month later, demonstrating the potential therapeutic benefit of combination therapy for NMOSD patients with high AQP4 IgG titers.

The failure of visual recovery in two patients (#3 and #4) was considered as the outcome of cumulative disability because of previous acute optic neuritis attacks. In addition, patient #3 received standardized treatment after 1 month of deterioration, severely limiting the treatment’s efficacy. It is difficult to determine whether a combination therapy of IVMP and efgartigimod may have been more effective for treating acute myelitis than optic neuritis. Vision loss is a significant contributor to the disease burden of NMSOD. Previous studies have shown that early initiation of high-dose IVMP within 7 days of an acute optic neuritis attack contributes to vision recovery in NMOSD [21] and is also more important than the timing or total times of PLEX and the total cycles of IVMP for visual prognosis [22].

Higher AQP4–IgG titers correlated with more severe attacks, including severe optic neuritis attacks [23]. Two patients (2/4) showed AQP4–IgG decrease along with symptoms improvement or EDSS decrease in this study. The decrease of antibody titers following treatment in the acute phase can be used as a favorable index for drug efficacy. PLEX was proven to be highly effective immunotherapy by washing out AQP4–IgG, complement, and proinflammatory factors [24]. However, intravenous infusion of efgartigimod and methylprednisolone overcomes the limitations of medical facilities, especially in areas without plasma exchange equipment, and avoids the risks of coagulation abnormalities, hypotension, hypocalcemia or catheter-related infections. More research is required to identify immunotherapy strategies that are safer, more effective, and more accessible.

In the matter of immunoglobulins, the reduction of IgG in three patients (#1–3) indicates that efgartigimod retains its biological function of rapidly degrading IgG in NMOSD patients. Previous studies have also shown that efgartigimod selectively cleans IgG without altering the levels of other immunoglobulins or serum albumin [9, 25, 26]. The reversible decrease in immumoglobin levels noted in this study suggests that combination therapy does not lead to permanent immunosuppression, thereby mitigating the potential risks of infection or other immunosuppressive side effects, which enhances patient safety. These results suggest that the safety profile of efgartigimod is favorable for managing acute NMOSD attacks.

Efgartigimod significantly reduces IgG levels by up to 50% after a single administration and further decreases IgG by an average of 75% with multiple doses [9]. A dose of 10 mg/kg achieved the maximum effect in reducing IgG levels in healthy volunteers, and high doses increased the probability of efgartigimod-related adverse events [9]. We administered a regimen of IVMP in combination with two infusions of efgartigimod, which was well tolerated. Extrapolation results from the best regimen of efgartigimod used for acute NMOSD attacks should be further investigated.

Previous research has shown that FcRn antagonists are successful in decreasing the levels of human serum IgG [9, 27] and serve as one of the novel treatment strategies for IgG autoimmune-mediated diseases. Therefore, efgartigimod is a promising therapeutic agent for acute NMOSD attacks via rapid clearance of the AQP4 IgG antibodies. However, the use of efgartigimod in acute NMOSD is still not widely reported.

The therapy of efgartigimod plus high—dose IVMP was well tolerated by four patients. The reported adverse events of efgartigimod were mild headache, fatigue, and back pain, without any allergic or serious events [9, 26]. In our study, hyperlipidemia, elevated blood pressure, or elevated intraocular pressure were possibly related to the effect of methylprednisolone. No infections or deaths occurred. In one study of batoclimab as an add-on therapy to methylprednisolone, the percentage of urinary tract infection was 33.3% (3/9) [11]. Yamasaki et al. reported one patient with urinary tract infection (1/73, 1.4%) with IVMP therapy [28]. The differences in research design, sample size, and data collection methods among various studies may lead to inconsistent results. The results of this study indicated that the infection rate was not higher with combination therapy compared to IVMP alone. However, more data is required to elucidate the statistical significance of the potential increased risk of infection.

This study has some limitations. First, data of dynamic monitoring AQP4–IgG titers was partly unavailable owing to the retrospective design and analysis in this study. Second, the sample size of the two groups was small given the rarity of the disease and the small number of NMOSD patients treated with efgartigimod in the acute phase. Third, high-dose of IVMP is still the first-line treatment for acute attacks in NMOSD; therefore, we focused predominantly on the synergistic effect of efgartigimod plus IVMP, which needs a true control group receiving a placebo or standard treatment for confirmation of results.

Efgartigimod is an FcRn inhibitor that is currently approved only for use in myasthenia gravis. However, it has also been attempted in various other neuroimmunological disorders. In the treatment of NMOSD, efgartigimod has shown promising effects. However, only case reports are available, and results from controlled clinical studies are still lacking. We compared the efficacy and safety between efgartigimod plus IVMP and IVMP alone for the first time, and our study findings provide important information about the usefulness and application of efgartigimod plus IVMP for NMOSD acute attacks in the real world.

## Conclusion

This retrospective study suggests that efgartigimod combined with IVMP might be associated with the positive effects of clearing AQP4 IgG-positive NMOSD patients in the acute phase. In our study, 50% patients in group 1 showed a reduction in EDSS compared to 14.3% patients in group 2 in the acute period. The average EDSS reduced more significantly when treated with a combination of efgartigimod and IVMP than IVMP alone. No serious side effects were reported, and efgartigimod therapy was well-tolerated with no discontinuation.

This study provides valuable data to clinicians. Efgartigimod concurrent with IVMP is safe and tolerable in the acute-phase of NMOSD.

## Data Availability

The data supporting the findings of this study are available in the figshare repository at 10.6084/m9.figshare.27636783.

## Abbreviations

AQP4: Aquaporin-4
CNS: Central nervous system
EDSS: Expanded disability scale score
FcRn: Neonatal Fc receptor
IA: Immunoadsorption
IgG: Immunoglobulin G
IVMP: Intravenous methylprednisolone
IVIG: Intravenous immunoglobulin
NMOSD: Neuromyelitis optica spectrum disorders
PLEX: Plasma exchange
